# The Presence of Methylation Quantitative Trait Loci Indicates a Direct Genetic Influence on the Level of DNA Methylation in Adipose Tissue

**DOI:** 10.1371/journal.pone.0055923

**Published:** 2013-02-19

**Authors:** Alexander W. Drong, George Nicholson, Åsa K. Hedman, Eshwar Meduri, Elin Grundberg, Kerrin S. Small, So-Youn Shin, Jordana T. Bell, Fredrik Karpe, Nicole Soranzo, Tim D. Spector, Mark I. McCarthy, Panos Deloukas, Mattias Rantalainen, Cecilia M. Lindgren, on behalf of the MolPAGE Consortia

**Affiliations:** 1 Wellcome Trust Centre for Human Genetics, University of Oxford, Oxford, United Kingdom; 2 Department of Statistics, University of Oxford, Oxford, United Kingdom; 3 Wellcome Trust Sanger Institute, Hinxton, United Kingdom; 4 Department of Twin Research, King's College London, London, United Kingdom; 5 Oxford Centre for Diabetes Endocrinology and Metabolism, University of Oxford, Oxford, United Kingdom; Institut national de la santé et de la recherche médicale, France

## Abstract

Genetic variants that associate with DNA methylation at CpG sites (methylation quantitative trait loci, meQTLs) offer a potential biological mechanism of action for disease associated SNPs. We investigated whether meQTLs exist in abdominal subcutaneous adipose tissue (SAT) and if CpG methylation associates with metabolic syndrome (MetSyn) phenotypes. We profiled 27,718 genomic regions in abdominal SAT samples of 38 unrelated individuals using differential methylation hybridization (DMH) together with genotypes at 5,227,243 SNPs and expression of 17,209 mRNA transcripts. Validation and replication of significant meQTLs was pursued in an independent cohort of 181 female twins. We find that, at 5% false discovery rate, methylation levels of 149 DMH regions associate with at least one SNP in a ±500 kilobase *cis*-region in our primary study. We sought to validate 19 of these in the replication study and find that five of these significantly associate with the corresponding meQTL SNPs from the primary study. We find that none of the 149 meQTL top SNPs is a significant expression quantitative trait locus in our expression data, but we observed association between expression levels of two mRNA transcripts and *cis*-methylation status. Our results indicate that DNA CpG methylation in abdominal SAT is partly under genetic control. This study provides a starting point for future investigations of DNA methylation in adipose tissue.

## Introduction

There is growing interest in the role of epigenetic factors in predisposition to common, and especially metabolic, diseases. Epigenetic effects are mitotically heritable alterations in gene expression that occur without alterations in the DNA sequence [1], but rather through molecular alterations such as histone modifications and DNA methylation.

These epigenetic marks are generally not inherited across generations [2], but DNA sequence variants that associate with methylation, known as methylation quantitative trait loci (meQTLs), have been found throughout the genome for a number of tissues [3]–[6]. Genetic effects on methylation thus provide a mechanism by which methylation patterns can be transmitted across generations.

Many studies on CpG methylation have been carried out in blood [7]–[9], but as methylation is highly tissue-specific [10], such study designs may not be relevant for metabolic traits. With epigenetic studies, tissue availability is generally a problem, but abdominal subcutaneous adipose tissue (SAT) represents one metabolically active tissue that is accessible and of relevance to obesity-related diseases [11]. In this study, we were interested in finding genetic variation that influences methylation status in abdominal SAT and in relating this to metabolic phenotypes, most particularly the cluster of obesity-related phenotypes described as metabolic syndrome (MetSyn) [12].

## Materials and Methods

### Samples and Phenotypes

We collected abdominal fat biopsies from the Oxford Biobank [13]. As part of MolPAGE the MolOBB case/control study for MetSyn (as defined by the International Diabetes Federation [IDF] [12]) involves 20 cases (10 male, 10 female) and 20 controls (10 male, 10 female). We excluded one male case and one male control from the analysis due to non-European descent and missing phenotype data, respectively, leaving 19 MetSyn case and 19 control samples for analysis (**Table 1**). The full dataset of 38 samples was included in the meQTL analysis. The study was approved by the National Research Ethics Service, Oxfordshire REC C (REC reference: 08/H0606/107) and informed consent in writing was collected from all participants.

**Table 1:** Participant characteristics (Primary Study).

Participant characteristics	All cases	All Controls	Male cases	Female cases	Male controls	Female controls
Sample size (N)	19	19	9	10	9	10
Age (years)	49±5	49±5	48±3	50±6	47±5	51±5
BMI	32.4±5.6	26.6±4.5	32.9±4.9	31.9±6.4	25±2.6	28±5.5
Waist (cm)	107±13	90±9	112±14	103±12	89±6	90±11
Hip (cm)	113±11	104±8	113±10	113±12	100±4	106±10
HDL (mmol/l)	1.03±0.26	1.54±0.25	0.85±0.13	1.19±0.25	1.36±0.21	1.68±0.18
TG (mmol/l)	2.3±1.6	1.1±0.3	2.6±1.8	1.9±1.3	0.9±0.2	1.2±0.3
Glucose (mmol/l)	5.6±0.5	5.1±0.3	5.7±0.5	5.6±0.5	5.2±0.4	5±0.3

Values are means ± standard deviation for each quantitative trait.

### Differential Methylation Hybridisation Data – Primary Cohort

We extracted DNA from abdominal SAT samples and determined the methylation status of 51,195 genome-wide probe set regions using Differential Methylation Hybridisation (DMH, Epigenomics AG, Germany). This method measures the quantity of methylated DNA after a methylation sensitive restriction digest by hybridisation onto a custom microarray (**Supplementary Information S1**). Validation by bisulphite sequencing and reproducibility of the DMH method have been confirmed in a previous study [14]. The DMH probe sets on the array cover 430 genes that are reported to be differentially methylated, 13,500 CpG-rich promoter regions and 13,650 CpG-rich regions within gene bodies across the genome [15].

Each biological sample was analysed in duplicate by hybridisation onto two DMH microarrays, which were randomized across three batches. Intensity values of each probe were averaged over the two duplicate chips per sample. A methylation score for each DMH region was calculated as the difference between the median log_2_-transformed probe intensities for each probe set and the median log_2_-transformed chip-internal signal control probe intensities [15]. Two additional chips containing 0% and 100% methylated DNA [15] were also analysed and used to calibrate the methylation score value to the 0% and 100% methylated DNA (range -1.20–2.75), applying methodology previously outlined [16]. Subsequently, we quantile-normalised the methylation score across individuals and excluded any DMH probe sets that contained SNPs with minor allele frequency (MAF) >5% in the 1000 Genotypes CEU Interim 2010 data [17]. This reduces allele bias introduced by the hybridisation step, but avoids exclusion of probe sets based on rare variants potentially not present in the sample.

### CpG Methylation Data - Replication cohort

The replication cohort consists of subcutaneous adipose samples from 181 female individuals (**Table 2**) from the MuTHER study [18], [19]. All subjects were recruited from the TwinsUK resource [20] previously shown to be comparable to population singletons in terms of disease-related and lifestyle characteristics [21]. The included adipose samples were randomised and genomic DNA was isolated using the NORGEN purification kit (Norgen Biotek Corporation, Canada) and quantified using picogreen. After a second round of randomisation, 750 ng of each DNA sample was taken for bisulphite conversion using the EZ-96 DNA Methylation Kit (Zymo Research) according to the protocol provided by the manufacturer. Methylation profiling of the bisulphite-converted samples was performed using the Illumina Infinium HumanMethylation27 BeadChip (Illumina 27k) that assays DNA methylation levels at 27,578 different CpG sites. The BeadChips were scanned using the IlluminaHiScan SQ scanner and raw data were imported to the BeadStudio 3.2 software (methylation module), which was used to extract the image intensities. The methylation score for each CpG was represented as a beta value (generated by the BeadStudio software) corresponding to the ratio of the intensity of the methylated bead type to the combined locus intensity and range from 0 (unmethylated) to 1 (fully methylated) on a continuous scale, which were filtered for quality (**Supplementary Information S1**).

**Table 2:** Participant characteristics (Replication Study).

Participant characteristics	
Sample size (N)	181
Age (years)	61.1±7.6
BMI	26.4±4.5
Weight (kg)	68.9±12.6
HDL (mmol/l)	1.9±0.5
TG (mmol/l)	1.2±0.6
Glucose (mmol/l)	5.1±0.7

Values are means ± standard deviation for each quantitative trait.

### Genotype Data – Primary Cohort

302,765 genetic markers from the 38 individuals were genotyped using the Illumina 317k BeadChip platform (Illumina Inc., USA) and filtered for (i) Hardy-Weinberg p-value ≤5.7x10^−7^ and (ii) minor allele frequency (MAF) >1% and genotype rate >99%, or >95% if MAF >5%. We imputed 11,116,176 SNPs using IMPUTE (v2.1.2) [22] and the 1000 Genomes Interim 2010 haplotypes as a reference panel [17]. Quality control was carried out by applying a filter for effective minor allele count >5% (2 * MAF * IMPUTE Info Score *38>5 [23], [24], resulting in 5,227,243 SNPs used for subsequent analyses. This corresponds to a minor allele count of 5. The IMPUTE Info Score multiplied by the sample size is the effective sample size, which is reduced by high genotype uncertainty [22].

### Genotype Data – Replication Cohort

Genotyping of the 181 replication samples was performed as part of the TwinsUK dataset (N ∼ 6000) with a combination of Illumina arrays (HumanHap300, HumanHap610Q, 1M-Duo and 1.2MDuo 1M). Intensity data for each of the three arrays were pooled separately (with 1M-Duo and 1.2MDuo 1M pooled together) and the Illumina calling algorithm assigned genotypes. No calls were assigned if the maximum posterior probability was less than 0.95.

After quality control (**Supplementary Information S1**) imputation was performed using the IMPUTE software package (v2.1.1.10) using two reference panels, P0 (1000 Genomes haplotypes, 2011 Phase I (interim) release, EUR) and P1 (the combined HumanHap610k and 1M reduced to 610 k SNP content) [17]. The SNP positions were mapped from b36 to b37 according the UCSC Feb. 2009 (GRCh37/hg19) assembly. Post-imputation, SNPs were filtered at a MAF >5% and IMPUTE info value of >0.8.

### mRNA Expression Data – Primary Cohort

We profiled mRNA expression on the Affymetrix human GeneChip HGU133 Plus 2.0 array (Affymetrix, USA), covering the transcription level of 17,209 Ensembl-annotated genes in 31 of the 38 abdominal SAT samples with data for methylation and genotypes. Using a custom chip definition file as described by Dai et al. [25], we grouped probes into probe sets corresponding to Ensembl annotated genes. In our subsequent analyses, we quantified mRNA expression as the robust multi-array average (RMA) without background correction, which is a summary measure of quantile-normalised, log_2_-transformed probe intensities [26].

### Phenotype Association

To test for association of DNA methylation with MetSyn, we applied linear models with the *limma* R package [27] using the Methylation score [16] of the 27,718 DMH probe sets that passed filtering as the response and case/control status (adjusting for age and gender) as the predictor variable. Similarly, we tested for association of either BMI or age with DMH methylation score as the response adjusting BMI for age and gender, and age for BMI and gender. Lastly, we analysed association of gender with methylation score for the autosomes only, adjusting for BMI and age. For each phenotype, a 95% confidence interval was calculated by performing 1000 permutations of the methylation scores.

### Cis-meQTL Association – Primary Study

We carried out a *cis*-meQTL association analysis using 29,441 filtered DMH regions, where we tested SNPs in a ±500 kb region around the probe set for association in a linear additive model using PLINK [28] (**Supplementary Information S1**). We accounted for genotype uncertainties by using an allelic dosage model [28], adjusting for age, gender and case/control status for MetSyn [4], [5]. We corrected for multiple testing using the *qvalue* package in R [29] and applied a false discovery rate (FDR) threshold of 5%.

### Cis-meQTL Association – Replication Study

To replicate any *cis*-meQTL findings from the DMH primary cohort, we first assessed how many probes on the Illumina 27 k array were located within a 1 kb distance of DMH probe sets with significant meQTLs in the primary DMH cohort. This distance was chosen because of the high correlation of CpG methylation within 1 kb observed in previous studies [4], [10]. Of the 149 DMH probe sets with meQTL, 27 had Illumina 27 k within 1 kb, and for 19 of these probes there were also SNP genotype data for the lead SNP or a SNP in high linkage disequilibrium (LD, r2>0.8) with the lead SNP. First, to test for replication, we used a one-tailed t-test in the direction of the original association, using a Gaussian null distribution as an approximation to the true null distribution (a t-distribution with 176 degrees of freedom). Second, to test for any additional associations of the 19 probes with *cis*-SNPs (MAF >5%, info >0.8) in a ±500 kb region we applied a linear mixed effects model in R [29], using the lmer() function in the lme4 package, fitted by maximum-likelihood. The models were adjusted for both fixed (age, batch, concentration after bisulphite conversion (BSC) and BSC efficiency) and random effects (family relationship and zygosity). A likelihood ratio test was applied to assess the significance of the SNP effect. The p-value of the SNP effect in each model was calculated from the Chi-square distribution with 1 degree of freedom using -2 log(likelihood ratio) as the test statistic.

### mRNA Expression Association

Subsequently, to assess the effect of CpG methylation on mRNA expression, we matched each DMH probe set that was significantly associated with a meQTL SNP at the 5% FDR level with the mRNA transcript of which the transcription start site (TSS) was closest in distance. The rationale for this approach is that CpG methylation represses transcription at gene promoters [4]. We fitted a linear model with expression level as the response variable and DMH probe set methylation score as the predictor, adjusting for age, gender and MetSyn case/control status. A similar analysis was carried out on all 27,718 DMH probe sets.

We tested the meQTL SNPs for *cis*-eQTL association with transcripts previously matched to the DMH regions under genetic control. Analogous to the meQTL analysis, we used a linear additive model with allelic dosage as a predictor, while adjusting for age, gender and case/control status for MetSyn. We applied an FDR threshold of 5% to all analyses.

### Text Mining and Pathway Analysis

First, we used DAPPLE [30] to investigate protein-protein interactions between the gene products of genes with meQTLs. As an input, we used the 149 meQTL SNPs and the default settings of 1,000 permutations and a common interactor binding degree cut-off of 2. By default, the gene regulatory region is defined as 50 kb up- and downstream of each transcript [30].

Second, for the gene pathway analysis, we assigned each meQTL SNP to an Ensembl-annotated gene with closest TSS. This was used as input for a gene set enrichment analysis (GSEA) using MAGENTA [31]. The software calculates gene scores from GWAS SNP p-values [31]. We used the default settings of gene boundaries 110 kb upstream and 40 kb downstream of each gene and 10,000 permutations were used to calculate the GSEA p-value cut-off. We tested for enrichment of genes assigned to meQTL hits in sets of genes highly associated with six phenotypes: (i) BMI [23], (ii) waist-hip ratio (WHR) adjusted for BMI [24], (iii) total cholesterol (TC), (iv) triglycerides (TG), (v) high-density lipoprotein (HDL), and (vi) low-density lipoprotein (LDL) [32]). These trait association data were derived from large GWAS datasets covering over 2 million SNPs.

Third, to detect whether the meQTL hits in SAT are enriched for associations previously found by GWAS, we carried out a permutation test (10,000 permutations) using a rank-sum statistic, where 149 random SNPs matched for MAF ±0.05, distance to genes ±500 kb and local recombination rate ±1 cM/Mb were chosen in each permutation.

Fourth, we tested whether those genes that overlapped *cis*-meQTL probe sets were statistically over-represented at each term in the GO database [33], relative to the number expected to occur by chance (a hypergeometric null distribution). The resulting p-values were adjusted at the 5% FDR level.

## Results

### Patterns of Abdominal SAT CpG Methylation

**Figure 1:**
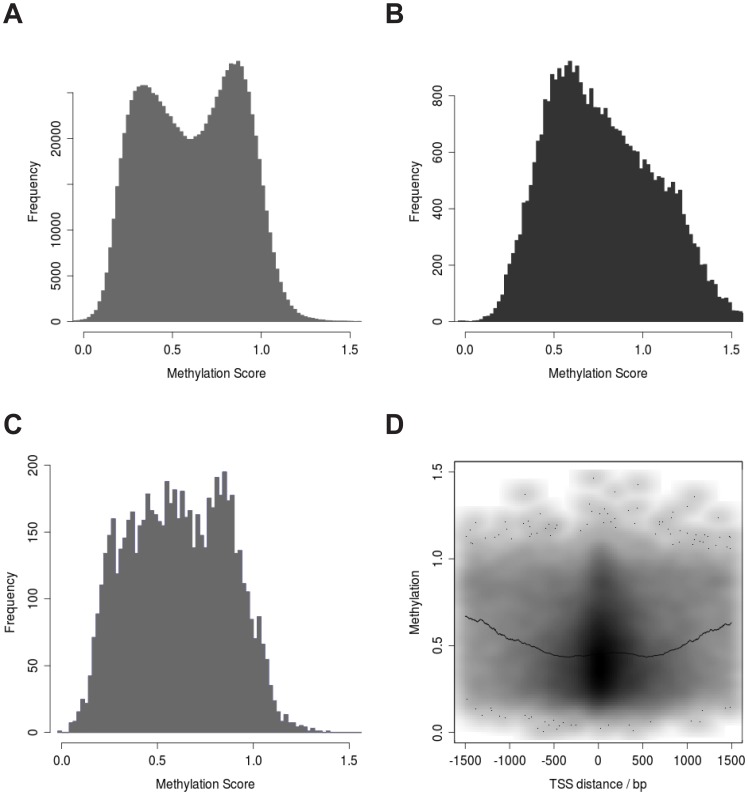
Patterns of CpG Methylation in abdominal SAT. A: DMH data show a bimodal pattern consistent with hypo- and hypermethylation. **B:** CpG sites close to TSS’s on the X chromosome are hemimethylated in females. **C:** CpG sites close to TSS’s of Imprinted Genes are hemimethylated **D:** Lower methylation is observed around TSS’s (black line shows 300 bp sliding window median methylation score).

We first set out to investigate whether the data measured by DMH fit with previously reported patterns of methylation [4], [5] and saw a convincing consistency with the expected patterns. Global methylation patterns measured by DMH in our study showed bimodality, with a “hypomethylated mode” at lower and a “hypermethylated mode” at higher methylation score (**Figure 1A**). We observed a slightly higher occurrence of hypermethylation in our study compared to what has been previously reported for the Illumina 27 k array [4], which can be explained by the difference in targeting of differentially methylated regions between the microarray designs. While the Illumina 27 k array probes are mainly located in promoter regions, the DMH array also targets a larger number of intra- and intergenic regions: 9,253 of the 12,500 genes targeted by the DMH array also are probed by the Illumina 27 k array (74.0%) [4], [5], only 3,629 probed sites directly overlap (13.1%). As expected, we found that the CpG sites on the X chromosome are hemimethylated (methylation score 0.3–0.7) [4] in females (**Figure 1B**). Also, the CpG sites close to the TSS of imprinted genes [34], were approximately hemimethylated (**Figure 1C**) and lower methylation was observed around transcriptional start sites (**Figure 1D**) [4], [10].

### Association of CpG Methylation with Metabolic Syndrome and Other Phenotypes

We then investigated whether the DMH methylation score at each probe set associated with MetSyn case/control status or BMI. At the 5% FDR level, we found no DMH probe sets that were significantly associated with MetSyn or BMI and the resulting p-value distribution remained within a 95% confidence interval generated by 1000 permutations of the methylation scores **(Figure S1A, S1B**).

**Figure 2:**
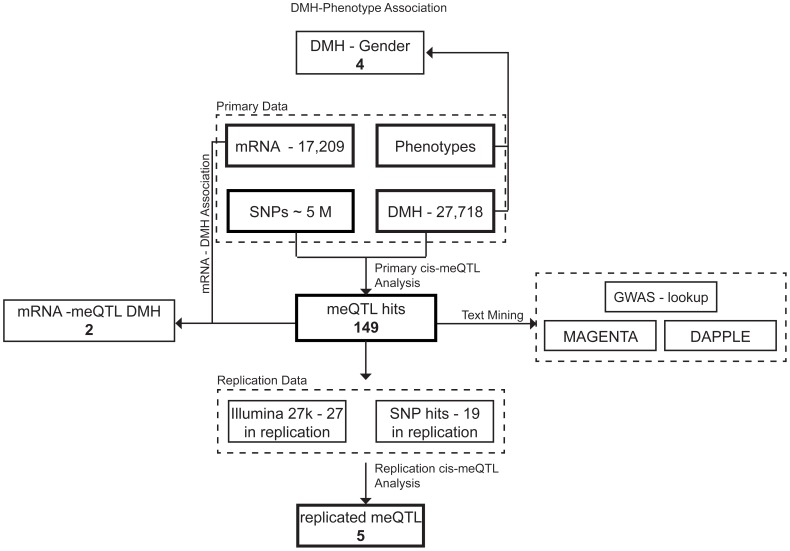
Flowchart showing the analysis pipeline. **Top:** Association of DMH Methylation Score with phenotypes. **Bottom:** Primary *cis*-meQTL association study, followed by replication study. **Left:** Association of DMH probe sets with significant meQTLs with mRNA expression. **Right:** Text mining of meQTLs significant in the primary study.

Additionally, we tested for association of methylation score with age and gender, excluding sex chromosomes in the latter case. We observed an association signal of gender with methylation score (**Figure S1C**) and four autosomal DMH probe sets significantly associated with gender at the 5% FDR level (**Figure 2**). No association of methylation score with age was detected (**Figure S1D**).

### Genome-wide meQTL Analysis

**Figure 3:**
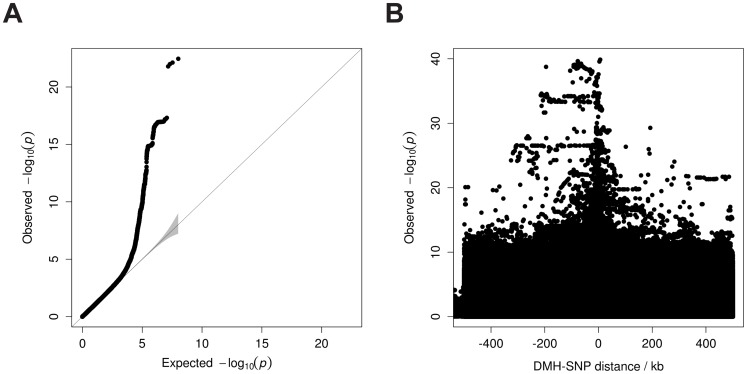
Genome-wide meQTL analysis. A: *Cis*-meQTL quantile-quantile plot showing enrichment of association signal. Grey bands correspond to 95% confidence intervals. **B:** Significant meQTL are located close to CpG sites.

First, we tested for *cis*-association of the methylation scores of the DMH probe sets with genetic variants (**Figure 2**, see (**Materials and Methods**). Our results showed an enrichment of low nominal p-values for SAT meQTLs (**Figure 3A**) and 149 meQTL sites were significant at the 5% FDR level (**Table S1**). These top meQTLs show a median distance of 80.7 kb between the DMH probe set and the lead SNP (**Figure 3B**, distance range 1 bp –499 kb), which is similar to the SNP- CpG distance of 81 kb reported by Gibbs et al. in brain tissue [5], despite the different platforms used between the studies.

Of the 149 significant DMH probe sets with meQTL SNPs, 19 have a corresponding Illumina 27 k CpG probe (that is located within 1 kb, see (**Materials and Methods**) and the same SNP, or a proxy SNP (r^2^>0.8), available (**Figure 2**). We analysed these in an independent sample of 181 abdominal SAT biopsies [19]. Five out of these 19 meQTL SNPs significantly associated with methylation levels in this replication sample (one-tailed test at the 5% level of significance, with the direction of significance being as identified in the primary study **Table 3** **, S2**, **Figures 3** **,** **4** **,** **5**). The replication rate was significantly higher than expected by chance (binomial p-value = 2.01×10^−3^). We then tested the 19 Illumina 27 k probes for associations with all SNPs within a ±500 kb *cis*-region. We found that only four probes for which the primary SNP replicated associated at the 5% FDR level with other nearby SNPs in the replication data (**Table S4**). In one case, the meQTL SNP was the same in the primary and replication study. A conditional analysis revealed that the weaker association signal of the primary and the replication study disappeared when adjusting for the SNP more strongly associated in either study (**Table S5**). This means that in some cases, there was an independent stronger *cis*-meQTL in the replication than the one detected in the primary study, where the difference was potentially caused by heterogeneity between the two sample populations or stochastic effects. In all cases the top SNP from the replication study was also significantly associated with methylation in the primary data (**Table S5**), which supports the replication.

**Figure 4:**
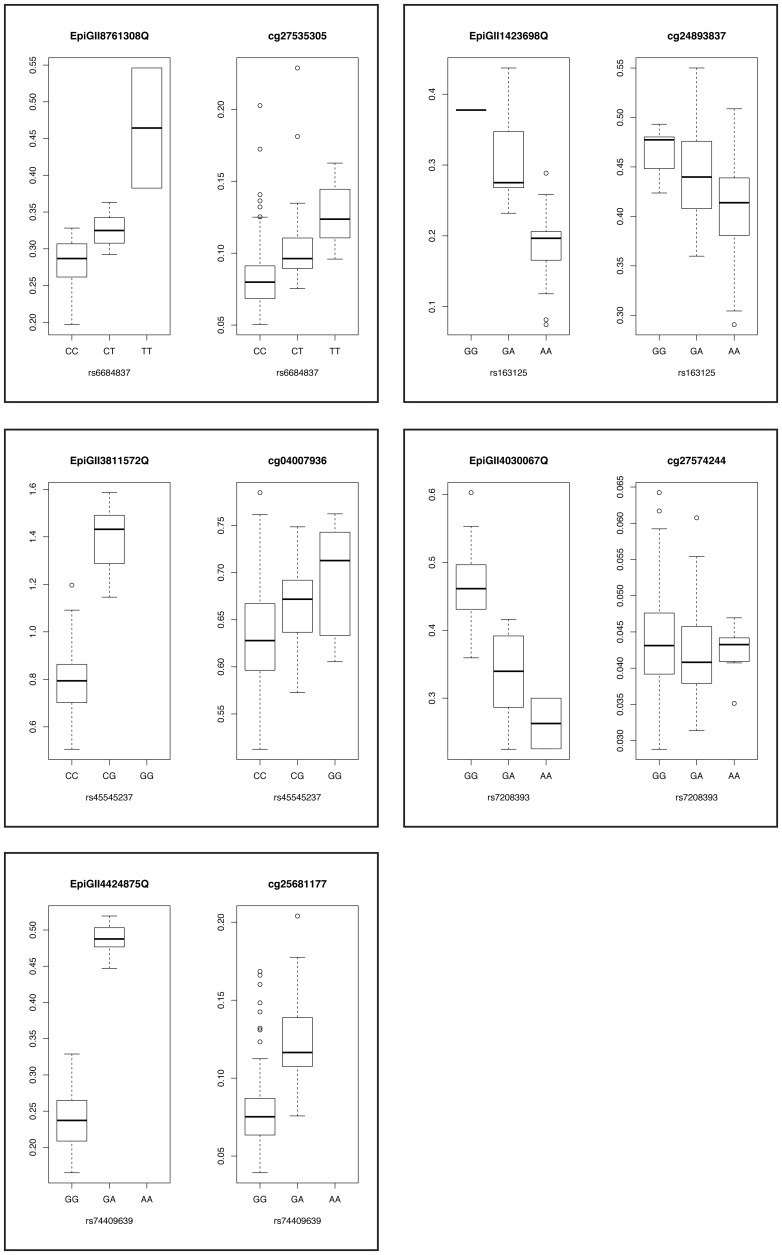
Boxplots showing methylation level plotted against genotype for the 5 replicated meQTLs in both the primary study (left panels) and replication study (right panels). All SNPs passed quality control filtering and association with methylation levels in both data sets.

**Figure 5:**
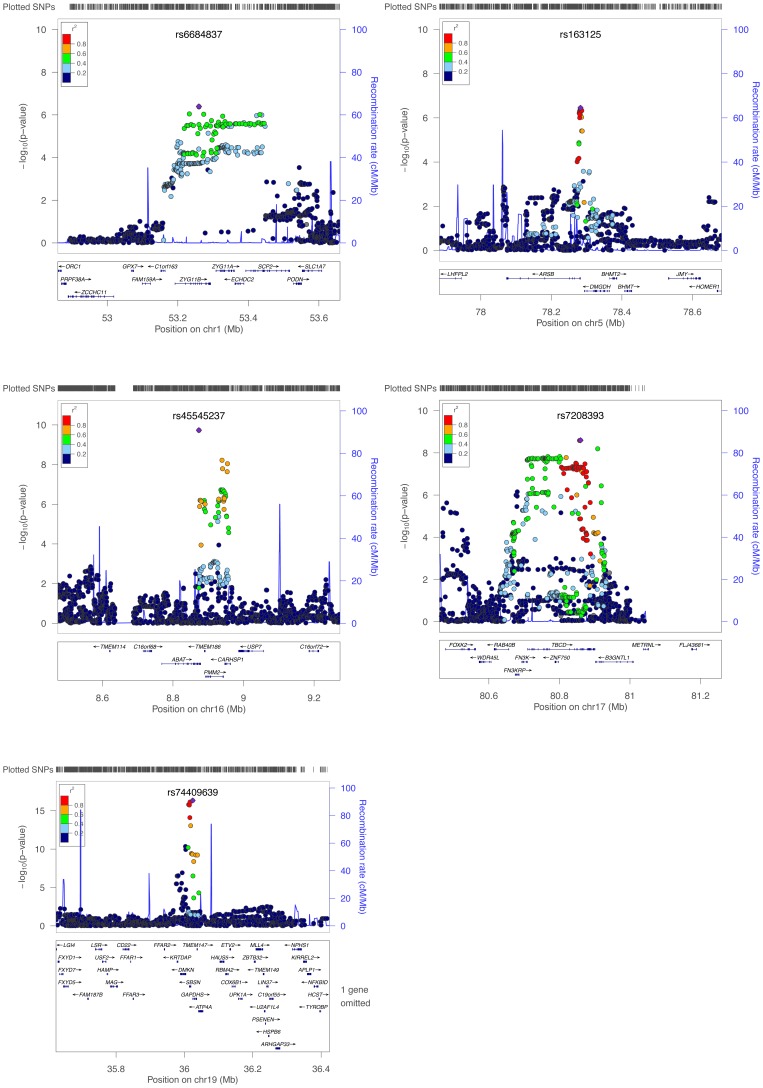
–log_10_ *P* values of the 5 replicated meQTLs against genomic position, with the top SNP indexed and indicated by a diamond. Estimated recombination rates are shown in blue, and SNP LD is given by colour as shown in the legends (LD data from 1000 Genomes Nov 2010 CEU genotypes).

**Table 3:** Replicated meQTL hits.

Primary Study								
Epigenomics ID^a^	Chr^b^	Probe Set Position^c^	SNP Position^d^	SNP rs ID		β_snp_^e^	s. e.(β_snp_)^f^	p-value^g^
EpiGII8761308Q	1	53392620–53392784	53259897	rs6684837		−0.0713	0.0113	4.05E–07
EpiGII1423698Q	5	78281698–78281893	78282670	rs163125		0.1148	0.0181	3.52E–07
EpiGII3811572Q	16	8954289–8954493	8873576	rs45545237		−0.7017	0.0775	1.85E–10
EpiGII4030067Q	17	80708321–80708551	80859844	rs7208393		0.1371	0.017	2.53E–09
EpiGII4424875Q	19	36024159–36024306	36024296	rs74409639		−0.2649	0.0166	4.72E–17
**Replication Study**								
**Illumina ID^h^**	**CpG Position^i^**	**β_snp_**	**s. e. (β_snp_)**	**one-sided p-value^j^**				
cg27535305	53393297	0.0203	0.0031	2.91E–11				
cg24893837	78282099	−0.0332	0.006	1.57E–08				
cg04007936	8954017	0.0352	0.007	2.47E–07				
cg27574244	80709357	−0.0018	0.0008	1.22E–02				
cg25681177	36024439	0.0484	0.0054	2.68E–19				

a) ID of probe set on DMH array,

b) chromosome.

c) genomic coordinates of probe set in build37.

d) genomic coordinates of SNP in build37.

e) coefficient of SNP effect.

f) standard error for the SNP effect.

g) p-value for the SNP effect.

h) ID of probe on Illumina 27k array,

i) genomic coordinates of CpG probed in build37.

j) one-sided p-value for the SNP effect in the direction of the original association.

Further, we investigated whether the meQTLs found in our study are also meQTLs reported in other tissues (i.e. LCLs or brain tissue) [4], [5]. We could investigate 27 of our 149 probe set- SNP tests, but we do not find any overlap between our meQTL associations found and the results from the HapMap LCLs [4]. Amongst the significant meQTLs reported by Gibbs *et al.* in brain tissue [5], we saw two of 887 probes (cg24893837 [*ARSB*], cg25681177 [*GAPDHS*]) that associate with the same SNP in our data (FDR <5%). One additional probe (cg04007936 [*CARHSP1*]) was also under significant genetic regulation although the SNP associated is independent (r2<0.1) of the lead SNP found in our study. In the latter case, this could indicate a general tendency to genetic control of methylation in this region or tissue specific differences.

### Association of mRNA Expression with CpG Methylation

We then investigated the association of DMH methylation scores with *cis*-mRNA expression of the transcript with the closest TSS. Firstly, when taking all 27,718 DMH probe sets into account no associations remained significant at the 5% FDR level (**Figure S2A).** We then considered only those 149 probe sets for which meQTLs existed and observed an association signal between methylation levels at DMH probe sets and mRNA expression (**Figure S2B**). In general, highly correlated associations between DMH probe sets and nearby transcripts tended to occur when the distance between the methylation site and TSS was short (**Figure S2C**). Results indicated that expression of two out of 149 mRNA transcripts were significantly associated with DMH methylation score at the 5% FDR level: EpiGII2424610Q (*TNFRSF11B*, Tumor necrosis factor receptor superfamily member 11B), and EpiGII8555714Q (*GOT1*, Aspartate aminotransferase). Distances between TSS and the correlating DMH probe set were 324 kb and 13 kb, respectively. In both cases, the correlation between DMH methylation and mRNA expression levels was negative.

To investigate whether the 149 meQTL SNPs are also eQTL SNPs, we tested association of 149 lead meQTL SNP genotypes with mRNA expression (**Figure S2D**). No associations were significant at the 5% FDR level. Thus, we did not observe control of gene expression by the meQTL SNPs.

### Gene Enrichment Analysis

We further investigated whether there are interactions between the protein products of the genes close to or overlapping the meQTL SNPs (**Figure 2** **,** see (**Materials and Methods**), which could potentially provide clues towards shared pathways by which the meQTLs act. We saw no enrichment in the observed direct or indirect interactions over the interactions expected by chance (p = 0.93 and p = 0.43) using DAPPLE [30], nor did we see an enrichment of genetic loci found by previous genome-wide association studies (GWAS) of the following phenotypes (i) BMI [23] (p = 0.69), (ii) WHR adjusted for BMI [24] (p = 0.71), (iii) TC (p = 0. 87), (iv) TG (p = 0.04), (v) HDL (p = 0.77), (vi) and LDL [32] (p = 0.32)) in the set of 149 lead meQTL SNPs after correction for multiple testing.

We then investigated whether the meQTLs are enriched in genes associated with the six phenotypes (i)-(vi) above. Using MAGENTA, we carried out a gene pathway enrichment analysis, assigning each DMH region to a gene, similar to the mRNA transcript assignment. For the six traits examined (see (**Materials and Methods**), only HDL associated genes (**Table S3**) had significant enrichment in the 149 genes tested (p = 7.40×10^−3^).

Lastly, we attempted to identify any other pathways in which the meQTLs could potentially be involved. With the FDR controlled at 5%, there were no significantly enriched GO terms.

### Data Availability

MolOBB gene expression data is available at ArrayExpress (E-MTAB-54), MolOBB genotype data is available at the European Genome-phenome Archive (EGAS00000000102) and MolOBB methylation data is available at http://www.well.ox.ac.uk/ggeu/PloSONE_Drongetal_MolOBB/.

## Discussion

Epigenetic marks, especially DNA methylation, play an important role in cellular differentiation and gene regulation in adipose tissue [10]. Moreover, genetic variants that influence DNA methylation may not only be a carrier for the inheritance of epigenetic marks [2], but also provide a biological mechanism by which SNPs act that have previously been shown to associate with disease phenotypes and/or downstream mRNA expression [4].

We first investigated whether DMH methylation associated with MetSyn status, BMI, age or gender. We find no associations of DMH methylation with MetSyn case control status or BMI. Changes in DNA methylation associated with BMI have been previously reported in a longitudinal study [35], but these effects might not have been observed in our study due to lower sample size. While we observed an association between methylation and gender, we find no association between methylation and age (**Figure S1D**), which is in line with a previous report [10]. The relatively modest sample size and narrow age distribution (SD = 5 years, **Table 1**) may impact power to detect age-related effects, which have been reported in the literature [4], [10], [36]. However, our main objective of this study was meQTL analysis, where effect sizes are expected to be larger, and where we are better powered to detect significant associations (**Supplementary Information S1**), as our results also indicate.

The results of our meQTL study indicate that there are a number of *cis*-meQTLs in abdominal SAT. Moreover, we observe that most *cis*-meQTLs act over a much shorter distance than the arbitrarily defined *cis* window of ±500 kb. In our replication study we attempted to replicate 19 out of 149 significant meQTL signals and find that five out of 19 DMH probe sets that reached significance in the primary study are also found to be significantly associated and directionally consistent in the replication study. The associations of methylation with SNPs in these five regions are consistent throughout the datasets, as each top *cis-*meQTL SNP within a ±500 kb region also is an meQTL in the other data set (**Table S5**). As methylation was measured on a different platform, the replication study also provided validation of the DMH method used in the primary study. There are three main aspects between the two platforms that may have an effect on the replication rate: (i) targeting of single vs. multiple CpGs, (ii) imperfect correlation between the DMH probe sets and Illumina 27k probes (within 1 kb) used as methylation proxies and (iii) methodological differences between the two assay techniques. Different genotyping scaffolds have been used and we use very stringent quality control measures pre-imputation, which means that even though different chips are used, the filtering on the minor allele count we do only allow high quality data, and variants with low allele frequency will be filtered out (**Supplementary Information S1**).

Importantly, in a recent report investing imputation accuracies using different methods and sizes of European reference panels, it was shown that for common variants with a minor allele frequency >5% the imputation accuracy performs similarly well [37]. Given this we feel confident that our association results, both on direct genotypes as well as the imputation, are as robust as they can be to genotype errors. However, the fact that methylation associates with genetic variants consistently across the two studies despite these differences suggests that in these regions there is indeed genetic control of methylation in SAT.

No significant associations of the 149 meQTL SNPs with expression of *cis*-mRNA transcripts are found, but association cannot be ruled out due to the relatively low statistical power in this study. Using similar assumption as above, and adjusting for 149 tests, we have 80% power to detect SNP-mRNA associations explaining 41% of variation in expression (**Supplementary Information S1**). Again, we are limited by the small sample size to detect associations of this magnitude.

Despite the current understanding that promoter methylation acts as a suppressor of mRNA translation, the observation that many meQTLs do not influence mRNA expression is consistent with the study in brain tissues by Gibbs *et al.* [5]. Bell *et al.* [4], on the other hand observe a significant enrichment for eQTLs in the meQTL results. This shows that there is still a need for future studies to investigate the downstream biological effects of methylation and the role of meQTLs. Our results show a significant enrichment for meQTLs found in brain tissue [5], but not for those in HapMap LCLs [4], potentially due to the lower power in the latter study. Limited overlap between the DMH probe sets and the Illumina 27k microarray used in these studies, as well as tissue-specific differences, may explain the low overlap between the study results. Further research could reveal the true degree of meQTL tissue specificity by using larger sample sizes and consistent assay techniques.

We find that two *cis*-mRNA’s significantly associated with meQTL DMH probe sets, encode for protein products that have previously been implicated in type 2 diabetes and MetSyn. *TNFRSF11B*, also known as *RANKL*, was previously characterized as an extracellular negative regulator of osteoprotegrin, which acts as a decoy receptor when secreted [38]. A number of studies have found that both osteprotegrin and this TNF-superfamily protein have elevated serum levels in type 2 diabetes patients [39], [40], and MetSyn [41]. *TNFRSF11B* is normally secreted by osteoblasts [38]. *GOT1*, also known as *AST1*, is a liver transaminase that plays a role in amino acid metabolism, the urea cycle and the Krebs cycle [42]. The *GOT1* gene promoter has been shown to be regulated by glucocorticoids, cAMP and insulin [43]. However, a role of these proteins in SAT has not been hypothesized or investigated previously.

Overall, we show for the first time that meQTLs are present in adipose tissue. This indicates a direct genetic influence on DNA methylation and also an indirect influence on the general molecular phenotype of adipose tissue. Defining the genetic influence on both gene expression and CpG methylation in abdominal adipose tissue can help towards characterising this type of tissue and understanding molecular pathways associated with obesity.

## Supporting Information

Figure S1 **Association of CpG methylation with metabolic syndrome and other phenotypes. A:** Association of methylation score with metabolic syndrome case/control status (linear regression). **B:** Association of methylation score with BMI. **C:** Association of methylation score with gender, chromosomes 1–22 only **D:** Association of methylation score with age. Grey bands correspond to 95% confidence intervals calculated by 1000 permutations of sample labels.
(TIF)

Figure S2 **Association of mRNA expression with CpG methylation. A:** Analysis of 27,718 DMH probes for association with downstream transcripts expression levels **B:** Analysis of 149 DMH probes with meQTLs for association with downstream transcripts expression levels. **C:** -log_10_(p) values plotted against DMH probe set-TSS distance. **D:** Analysis 149 meQTL SNPs (top hit for each probe) for association with mRNA transcript expression levels. Grey bands correspond to 95% confidence intervals.
(TIF)

Table S1 Full list of significant SNP-DMH probe set pairs.
(XLS)

Table S2 All primary meQTL hits tested for replication.
(XLS)

Table S3 Gene set enrichment analysis results.
(XLS)

Table S4 500 kb cis-meQTL analysis of 19 probes in the replication study.
(XLS)

Table S5 Conditional analysis between top meQTL SNPs found in the primary and replication study.
(XLS)

Supplementary Information S1 **Supplementary methods and power calculations.**
(PDF)
