# Supplementary figures and images for: The Presence of Methylation Quantitative Trait Loci Indicates a Direct Genetic Influence on the Level of DNA Methylation in Adipose Tissue
Source: PLoS One. 2013 Feb 19;8(2):e55923. doi: 10.1371/journal.pone.0055923 (PMC3576415; doi:10.1371/journal.pone.0055923)

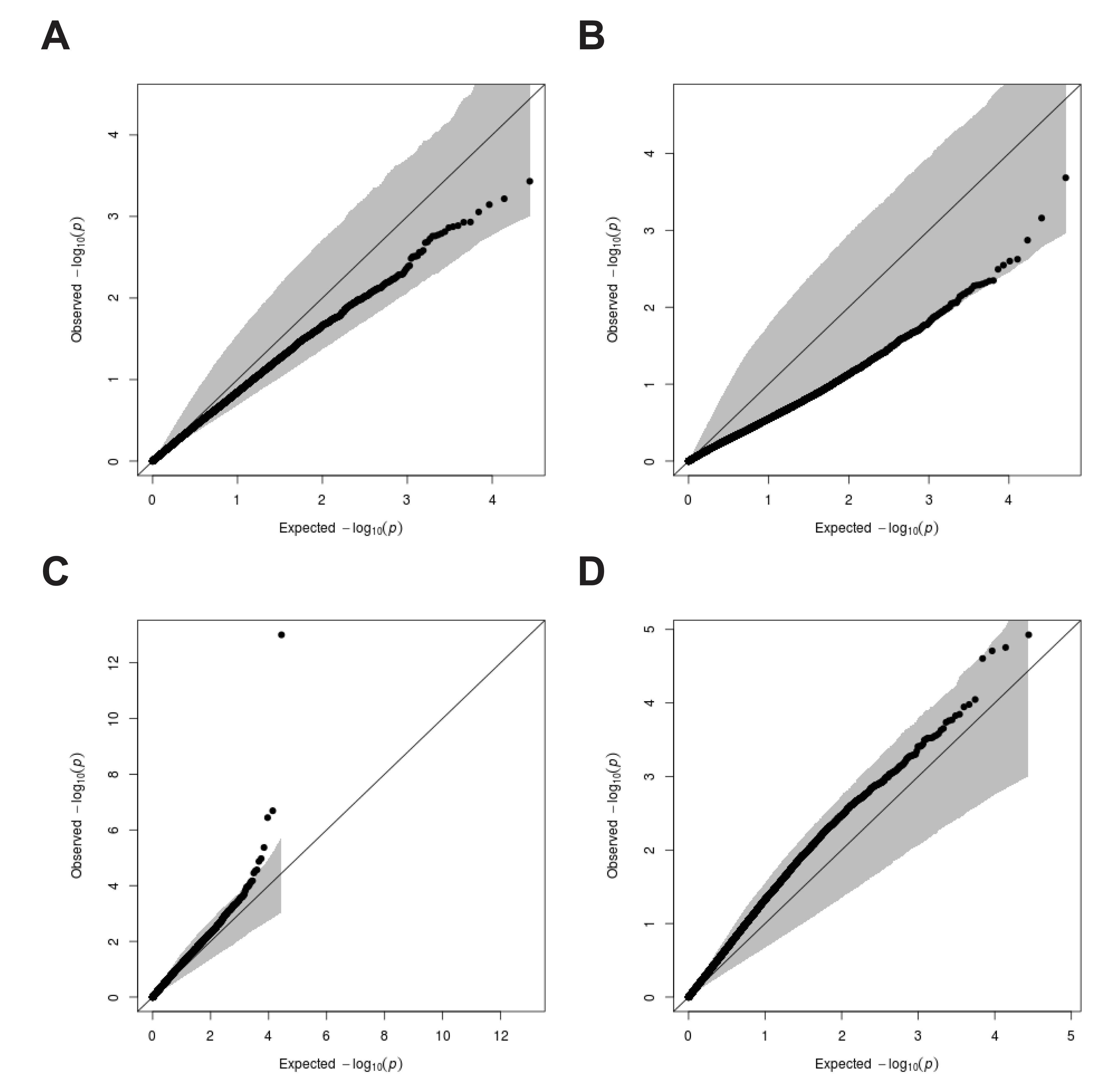

Supplement: Figure S1 — Association of CpG methylation with metabolic syndrome and other phenotypes. A: Association of methylation score with metabolic syndrome case/control status (linear regression). B: Association of methylation score with BMI. C: Association of methylation score with gender, chromosomes 1–22 only D: Association of methylation score with age. Grey bands correspond to 95% confidence intervals calculated by 1000 permutations of sample labels. (TIF) [file pone.0055923.s001.tif]

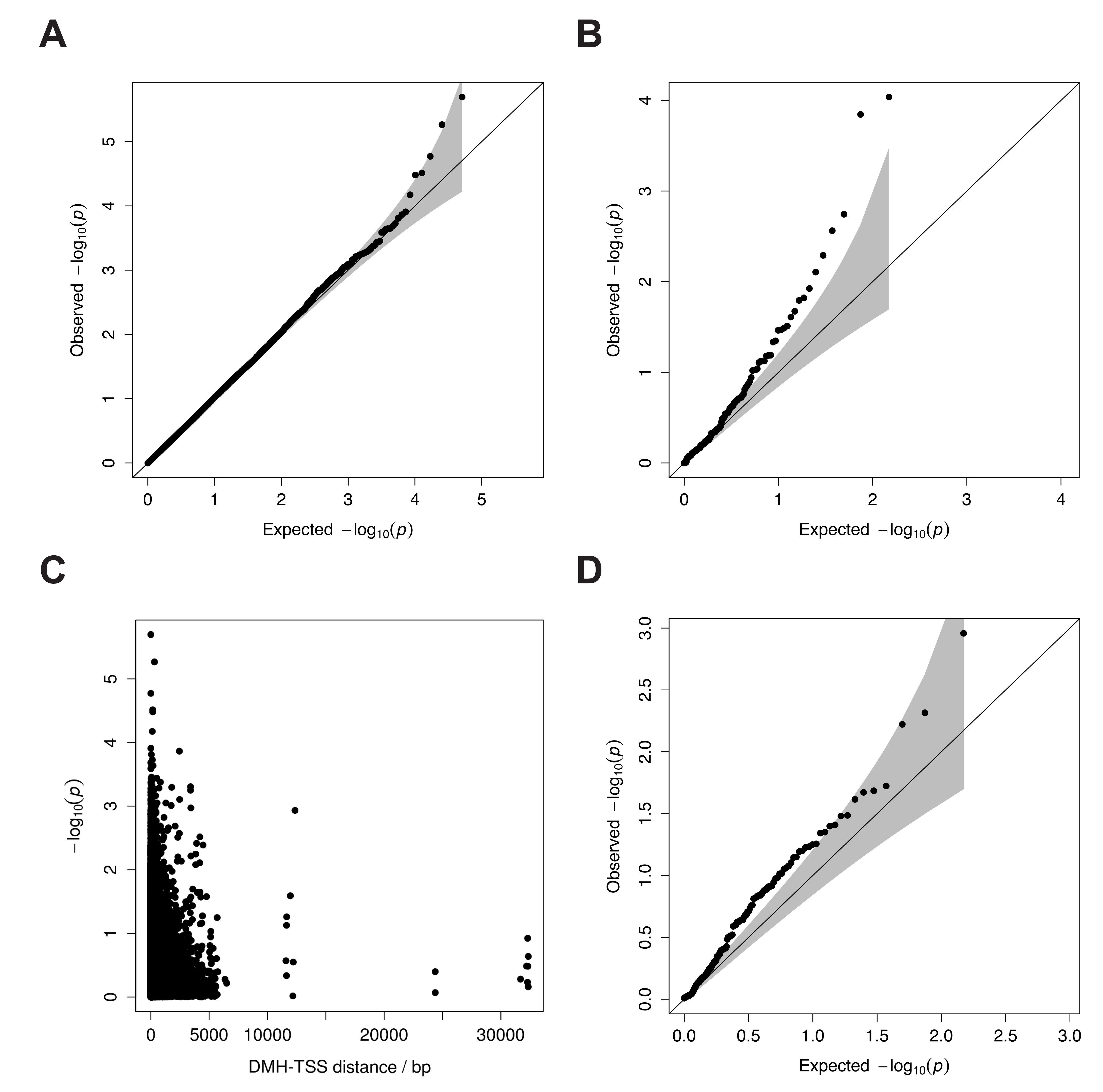

Supplement: Figure S2 — Association of mRNA expression with CpG methylation. A: Analysis of 27,718 DMH probes for association with downstream transcripts expression levels B: Analysis of 149 DMH probes with meQTLs for association with downstream transcripts expression levels. C: -log10(p) values plotted against DMH probe set-TSS distance. D: Analysis 149 meQTL SNPs (top hit for each probe) for association with mRNA transcript expression levels. Grey bands correspond to 95% confidence intervals. (TIF) [file pone.0055923.s002.tif]
