# Supplementary material for: The Presence of Methylation Quantitative Trait Loci Indicates a Direct Genetic Influence on the Level of DNA Methylation in Adipose Tissue
Source: PLoS One. 2013 Feb 19;8(2):e55923. doi: 10.1371/journal.pone.0055923 (PMC3576415; doi:10.1371/journal.pone.0055923)
Supplement: Supplementary Information S1 — Supplementary methods and power calculations. (PDF) [file pone.0055923.s008.pdf]

Supplementary Information

DMH Methodology

The DMH workflow was first described by Huang et al. [1]. Genomic DNA was digested with methylation unspecific restriction enzymes [2], with short fragments removed (<80 bp) in the purification step. Next, DNA oligomer adapters were ligated to restricted DNA fragments, which were then digested using four different CpG methylation specific restriction enzymes [2]. In a subsequent PCR step, only uncut (unmethylated) fragments were thus amplified as the digested (methylated) DNA does not have both 5' and 3' adapters for PCR primer binding [1]. PCR products were then hybridised to the custom-designed DMH chip based on the CustomSeq microarray (Affymetrix, USA).

CpG Quality Control – Replication Cohort

Quality control of the samples resulted in exclusion of seven samples including one sample with low bisulphate bisulphite (BS) conversion efficiency (i.e. BS control intensity values <4000) and six based on CpG coverage (requiring at least 95% coverage per sample), using the Beadstudio p-values of detection of signal above background. Probes that failed (N = 1459) and were not reported by the BeadStudio software in at least one individual were discarded.

A singular value decomposition (SVD) was performed to determine the nature of the largest components of variation in beta values as previously described [3]. By correlating the top ten principal components to known experimental factors it was shown that beadchip, BS conversion efficiency (as assessed using the built-in BS conversion efficiency controls) and DNA input contributed significantly to the variation in beta levels and were together with age included as covariates in subsequent analysis.

SNP Quality Control – Replication Cohort

We applied similar exclusion criteria to each of the three datasets. Samples were excluded if (i) sample call rate < 98%, (ii) heterozygosity across all SNPs ≥2 SD from the sample mean; (iii) evidence of non-European ancestry as assessed by PCA

comparison with HapMap3 populations; (iv) observed pairwise IBD probabilities suggestive of sample identity errors. SNPs were excluded if (i) Hardy-Weinberg p-value < 10^{-6}, assessed in a set of unrelated samples; (ii) MAF < 1%, assessed in a set of unrelated samples; (iii) SNP call rate < 97% (SNPs with MAF ≥ 5%) or <99% (for 1% ≤ MAF < 5%).

We then merged the three datasets after performing pairwise comparison between datasets. Further samples and SNPs were excluded if (i) concordance at duplicate samples <1%; (ii) concordance at duplicate SNPs <1%; (iii) visual inspection of QQ plots for logistic regression applied to all pairwise dataset comparisons; (iv) Hardy-Weinberg p-value < 10−6, assessed in a set of unrelated samples; (v) observed pairwise IBD probabilities suggestive of sample identity errors.

Statistical Methods – Methylation / Phenotype association

We used the R *limma* package, a package for the efficient analysis of microarray data to fit the following linear models to test for association with Metabolic syndrome case/control status (Eq. 1), BMI (Eq. 2), Age (Eq. 3) and gender (Eq. 4):

$$y = \beta_o + \beta_{MetSyn}x_{MetSyn} + \beta_{age}x_{age} + \beta_{gender}x_{gender} + \varepsilon \text{ (1)}$$

$$y = \beta_o + \beta_{BMI}x_{BMI} + \beta_{age}x_{age} + \beta_{gender}x_{gender} + \varepsilon \text{ (2)}$$

$$y = \beta_o + \beta_{age}x_{age} + \beta_{BMI}x_{BMI} + \beta_{gender}x_{gender} + \varepsilon \text{ (3)}$$

$$y = \beta_o + \beta_{gender}x_{gender} + \beta_{BMI}x_{BMI} + \beta_{age}x_{age} + \varepsilon \text{ (4)}$$

where y is the methylation score at each probe set and the error term is $\varepsilon \sim N(0, \sigma^2)$ for each marker independently. The method in *limma* then shrinks the probe set specific variances towards a common expected variance and calculates a moderated t-statistic, and the corresponding p-value for H_0: $\beta_{predictor} = 0$ is then calculated. Subsequently, multiple testing was corrected for by applying a false discovery rate (FDR) threshold of 5% (using the *qvalue* package in R [4]).

Statistical Methods – Primary Cohort meQTL association testing

To test for association of methylation with each SNP in ±500 kb region around each of the 29,441 filtered DMH regions, we applied a linear additive model using as described in the PLINK manual. The allelic dosage for each individual i at each SNP marker g was calculated as

$$x_{g,i} = p_{1,i} + 2\,p_{2,i} \text{ (1)}$$

where $p_{1,i}$ and $p_{2,i}$ are the posterior probabilities for the imputed genotypes AB (heterozygote) and BB (homozygote for the alternative allele), respectively. We then regressed methylation status at each probe set y with the allelic dosage using the following linear model:

$$y = \beta_o + \beta_g x_g + \beta_{age} x_{age} + \beta_{gender} x_{gender} + \beta_{MetSyn} x_{MetSyn} + \varepsilon \text{ (2)}$$

where the error term is $\varepsilon \sim N(0, \sigma^2)$ for each marker independently. We then calculated a p-value for H_0: $\beta_g = 0$ by calculating the Wald statistic $\frac{\beta_g^2}{se(\beta_g)^2}$, where $se(\beta_g)^2$ is the standard error in the estimate of β_g. This was then compared to a chi-squared distribution (Wald test). Multiple testing was corrected for by applying a false discovery rate (FDR) threshold of 5% (using the *qvalue* package in R [4])

Statistical Methods – Replication Cohort

Similarly, we calculated the allelic dosage for each SNP marker in the replication and fitted the following linear mixed-effect model allowing for twin relatedness:

$$y = \beta_o + \beta_g x_g + \beta_{age} x_{age} + \beta_c x_c$$

$$+\beta_{batch} x_{batch} + \beta_{BSC} x_{BSC} + Z_{family} U_{family} + Z_{zygosity} U_{zygosity} + \varepsilon \text{ (3)}$$

where x_c is the concentration after bisulphite conversion, x_{batch} is the batch, x_{BSC} the bisulphite conversion efficiency measured by the array-internal control, the random effect U_{fam} common to a pair of twins and the random effect U_{zyg}, which is shared by a pair of monozygotic twins but not by a pair of dizygotic twins [5].

We then calculate $\frac{\beta_g}{se(\beta_g)}$ and compare this to a one-tailed t-test in the direction of the original association, using a Gaussian null distribution as an approximation to the true null distribution (a t-distribution with 176 degrees of freedom)

Power Calculations

In this study we calculated the effect sizes that were detectable with our sample size, N = 38, and a significance level specified in the multiple-testing setting. We used a conservative Bonferroni correction strategy, meaning that if we test 27,578 DMH probe sets the adjusted significance level is α = 0.05 / 27,578, i.e. α ~ 1e-6 (Figure A). At a fixed α of 1e-6 and 80% power we are able to detect MetSyn case/control associations down to a standardized effect size of 2.16. We further estimated the detectable effect size in units of methylation score for each probe set, since the standardized effect size is scale-free and the within-group standard deviation (SD) can vary across probe sets using the following approach: (i) We calculated the pooled within-group SD at each probe set; (ii) multiplied the pooled within-group SD at each probe set with 2.16 (see above) and (iii) and then calculated for the proportion of probe sets were we have 80% power to detect methylation differences between groups of 0.05 and 0.10 methylation score. In our data, we are thus powered at 80% to detect effect sizes of 0.05 differential methylation at 14 of 27,718 probe sets and 0.10 methylation score difference at 4123 of 27,718 probe sets, where 0.05 and 0.10 here refer to difference in methylation score (not statistical significance level) between cases and controls.

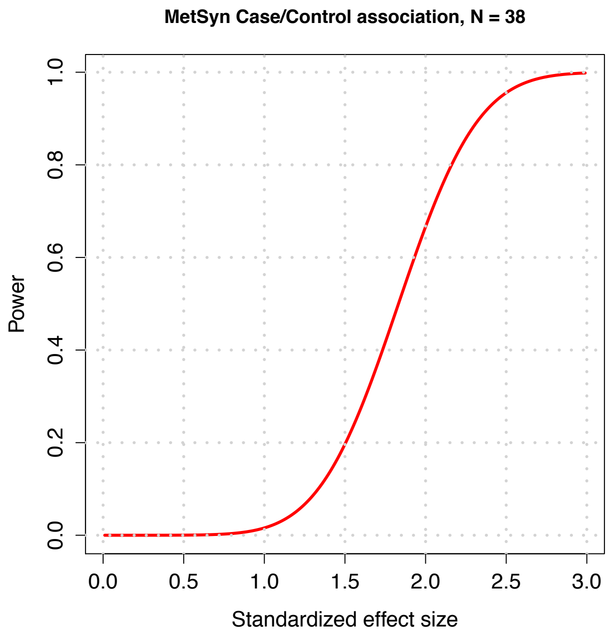

Figure A: Power calculation curve of power against standardised effect size, at fixed N=38 and alpha=1e-6.

Using the equivalent assumptions of α and power for BMI, which is a quantitative trait, we are powered to detect methylation-BMI associations explaining 57% of variation in BMI (Figure B, black curve). We are well aware that we are underpowered to detect subtle MetSyn and BMI effects in this relatively small study, which can explain the lack of detection of significant associations. However, our main objective of this study was meQTL analysis, where effects sizes are expected to be larger, and where we are better powered to detect significant associations, as our results also indicate.

Using similar assumption as above, and adjusting for 149 tests, we have 80% power to detect SNP-mRNA associations explaining 41% of variation in expression (Figure B, blue curve). Again, we are limited by the small sample size to detect associations of this magnitude.

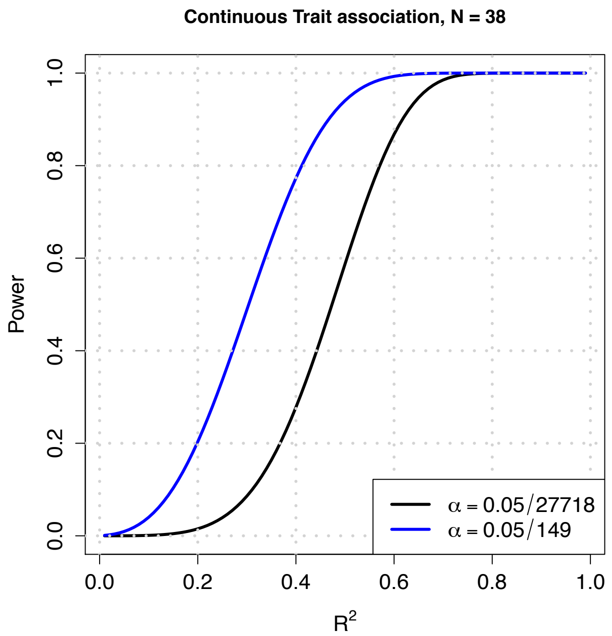

Figure B: Power calculation curve of power against percent variance explained, at fixed N=38 and two fixed alpha levels. The curves arise from differences in multiple testing.

References

1. Huang, T.H., M.R. Perry, and D.E. Laux, *Methylation profiling of CpG islands in human breast cancer cells.* Hum Mol Genet, 1999. **8**(3): p. 459-70.
2. Fassbender, A., et al., *Quantitative DNA methylation profiling on a high-density oligonucleotide microarray.* Methods Mol Biol, 2010. **576**: p. 155-70.
3. Teschendorff, A.E., et al., *An epigenetic signature in peripheral blood predicts active ovarian cancer.* PLoS One, 2009. **4**(12): p. e8274.
4. R Devlopment Core Team, *R: A language and environment for statistical computing.* R Foundation for Statistical Computing Vienna Austria ISBN, 2011. **3**(10).
5. Visscher, P.M., et al., *Assumption-Free Estimation of Heritability from Genome-Wide Identity-by-Descent Sharing between Full Siblings.* PLoS Genet, 2006. **2**(3): p. e41.
